# Research on Autonomous and Collaborative Deployment of Massive Mobile Base Stations in High-Rise Building Fire Field

**DOI:** 10.3390/s23187664

**Published:** 2023-09-05

**Authors:** Ke Li, Chen Huang, Jiaping Liang, Yanbin Zou, Biao Xu, Yao Yao, Yang Zhang, Dandan Liu

**Affiliations:** 1 Department of Electronic Engineering, Shantou University, Shantou 515041, China; ericlee@stu.edu.cn (K.L.); 21chuang@stu.edu.cn (C.H.); 21jpliang@stu.edu.cn (J.L.); ybzou@stu.edu.cn (Y.Z.); xubiao@stu.edu.cn (B.X.); 2 Naval Research Academy, Beijing 100161, China; 3 Yancheng Institute of Technology, Yancheng 224051, China; ldd@ycit.edu.cn

**Keywords:** swarm intelligence, trajectory planning, fire rescue, autonomous deployment, collaborative positioning

## Abstract

High-rise building fires pose a serious threat to the lives and property safety of people. The lack of reliable and accurate positioning means is one of the main difficulties faced by rescuers. In the absence of prior knowledge of the high-rise building fire environment, the coverage deployment of mobile base stations is a challenging problem that has not received much attention in the literature. This paper studies the problem of the autonomous optimal deployment of base stations in high-rise building fire environments based on a UAV group. A novel problem formulation is proposed that solves the non-line-of-sight (NLOS) positioning problem in complex and unknown environments. The purpose of this paper is to realize the coverage and deployment of mobile base stations in complex and unknown fire environments. The NLOS positioning problem in the fire field environment is turned into the line-of-sight (LOS) positioning problem through the optimization algorithm. And there are more than three LOS base stations nearby at any point in the fire field. A control law which is formulated in a mathematically precise problem statement is developed that guarantees to meet mobile base stations’ deployment goals and to avoid collision. Finally, the positioning accuracy of our method and that of the common method were compared under many different cases. The simulation result showed that the positioning error of a simulated firefighter in the fire field environment was improved from more than 10 m (the positioning error of the traditional method) to less than 1 m.

## 1. Introduction

Due to the high land price, limited land resources, and the rapidly growing economy’s demand for landmark buildings, high-rise and super high-rise buildings have become one of the effective means to address the space demand of urbanization and economic growth [1,2,3]. China is home to 5 of the world’s top 10 skyscrapers [4]. Statistics show that the number of high-rise and super high-rise buildings in China have been in the forefront in the world for eight consecutive years [5,6,7]. Although super high-rise buildings bring people enjoyment in life and greatly save space, they also bring people many problems. Fire is one of the most serious problems. According to statistics, urban high-rise building fire accidents can account for more than 90% of all fire accidents [8,9], and it is difficult to rescue and safely evacuate, which has brought huge losses to the national economy and people’s lives and property.

The biggest problem in the high-rise building fire rescue and evacuation of people in distress is positioning. Because the global positioning system (GPS) cannot be used in the indoor environment and the positioning base station cannot be deployed in advance, the positioning system in a high-rise building fire rescue has to face the impact of the non-line-of-sight environment, which greatly affects the positioning accuracy and real time. Firefighters and people in distress cannot obtain their exact positions in real time, which leads to the very low efficiency of rescue and evacuation. Therefore, the ultra-wide band (UWB) positioning system is the best choice in this case. The accuracy of the UWB base station coordinates affects the positioning accuracy of firefighters directly. So, obtaining the accurate position of the UWB base stations is very significant for the firefighters’ safety. But traditional methods are very difficult to obtain the accurate position of the base station in an environment with unknown obstacles [10]. It cannot meet the needs for the rapid deployment of UWB base stations.

Non-line-of-sight (NLOS) positioning means that there is at least one obstacle on the straight line between the base station and the positioning objective. Whereas line-of-sight (NLOS) positioning means that there is no obstacle on the straight line between the base station and the positioning objective. Non-line-of-sight (NLOS) positioning is much more difficult than LOS positioning so it has always been a bottleneck problem to improve the positioning accuracy in complex environments [11]. Obstacle obstruction will cause multipath effects, signal hysteresis, and other negative effects [12,13]. Up to now, no ideal solution has been found [14,15]. Targeting this bottleneck problem, a method for the autonomous deployment of positioning base stations based on swarm intelligence is proposed. The cooperation among unmanned aerial vehicles (UAVs) is used to achieve the autonomous coverage and deployment of the fire field, and the non-line-of-sight environment is converted into the line-of-sight environment, so as to achieve the accurate positioning of targets in the fire field environment.

Given a known environment without obstacles, the algorithms for the deployment of base stations are well studied [16,17,18]. The main practical motivation and application for the deployment of base stations in an ideal environment is to construct a communication network in the air to provide wireless signal coverage for the fire field [19,20]. In recent years, many scholars have applied machine-learning methods to solve control and path planning problems in multi-agent systems, such as deep learning, reinforcement learning, and deep reinforcement learning, and have achieved some success [21,22,23]. However, there are still many big challenges in solving the control problem of multi-agent systems in complex unknown closed environments, such as high-rise building fire fields. The multi-agent system used for fire environment detection has extremely high requirements for stability, reliability, environmental adaptability, lightweight, and so on. Machine learning cannot meet the application requirements for the detection of a high-rise building fire field because of the lack of interpretability and generalization.

Therefore, there has been very little work in the literature on how multi-UAV systems solve the positioning problem of NLOS in an unknown complex closed and GPS-denied environment with swarm UAVs. Existing works in the literature require robots equipped with sensors that are able to localize themselves precisely. The literature [24,25] focuses on using object-level features with both semantic and geometric information to model landmarks in the environment not need a prior constructed precise geometric map, which greatly releases the storage burden, especially for large-scale navigation. The algorithm is effective, but the deployment needs the accurate positioning information of UAVs that cannot meet the requirements for a high-rise building fire rescue. To the best of our knowledge, this is the most feasible approach for solving the precise positioning problem of a high-rise building fire rescue using GPS-denied swarm UAVs.

The main contribution and novel part in this paper is that it provides the approach for transforming the NLOS positioning problem into the LOS positioning problem in a 2D unknown complex environment with swarm UAVs and a host computer. There are two fundamental problems in the deployment of mobile base stations for coverage with communication constraints. The first problem is how to control the movement and behavior of multi-agent systems to realize a desired configuration. The second one is how to optimize the deployment in order to enhance the efficiency of the multi-agent system. A UAV lands and keeps static as a base station when UAV obtains the right position. Every UAV is equipped with a UWB positioning device to obtain the distance to adjacent UAVs and Zigbee equipment to send its own relative position information to other adjacent base stations and the host computer.

The rest of this paper is organized as follows: In Section 2, we formulate the problem and then define the sensing area of the UAV and the function of the sensing ability. The optimization method for the autonomous cooperative coverage deployment of base stations is presented in Section 3. The control law of multi-UAVs is given to make the coverage deployment operation feasible in complex environments in the presence of unknown obstacles and to ensure it can turn a non-line-of-sight environment into a line-of-sight environment. Section 4 demonstrates the effectiveness and feasibility of the proposed approach via computer simulations. Section 5 summarizes the paper and draws some conclusions.

## 2. Problem Formulation

To achieve precise target positioning in the fire field, there are two main problems to be solved in the optimization algorithm for the autonomous deployment of base stations in complex unknown environments.

1. How to realize the coverage deployment of mobile base stations in the fire field, and ensure that the target at any point in the area will be surrounded by at least three base stations nearby that can locate the target with LOS;
2. How to avoid collision when a large-scale multi-UAV system moves in a complex and unknown environment.

This becomes more difficult in the absence of GPS for navigation and positions. Without the global information, agents can only obtain local localization via a limited information exchange and estimate their own position or neighbor’s relative position based on the relative distance they measured.

### 2.1. Study Objective

In the process of solving the first problem, we set the different coverage values for the fire field according to the duration of the different area being detected and guided the UAV to move to the area where it is not effectively detected (the coverage value of the area is less than the effective coverage value).

### 2.2. Assumptions and Constrains

In this paper, a UAV is denoted by ***A***. Let *S* be a region that it is required to cover. Let *N* be the number of UAVs and let $p_{i}$ and $v_{i}$ denote the position and the velocity of UAV $A_{i}$, respectively, i∈I={1,2,3,…,N}. Each UAV $A_{i}$, i∈I, satisfies the following such kinematic equations of motion:

(1) $${\dot{p}}_{i}=v_{i},\;i\in I$$

Define the instantaneous coverage function $D_{i}$ as a continuous map that describes how effective a UAV $A_{i}$ senses a point p∈S. In this paper, we consider sensors with the following properties:

(1) The communication of a multi-UAV system is fully connected.
(2) The detection probability of the on-board sensor is 100%, that is, the detection probability of the sensor detection equipment has no effect on the results.
(3) Each UAV has a limited sensory domain $D_{i}(t)$ with a sensory range $R_{i}$. The sensory domain of each UAV is given by
  (2) $$D_{i}(t)=\{p\in S:||p_{i}(t)-p||\leq R_{i}\}$$
  Let the union of all coverage regions be denoted by $D(t)=\cup_{i\in S}D_{i}(t)$.
(4) Without the loss of generality, we consider the vision-based sensor for aerial detection may be modeled by following mathematical Formula (3). Each UAV’s sensor has a circular sensing symmetry about the position $p_{i}$, which is a practical property of a sensor in real applications. Within the sensing range of the UAV, each UAV has a peak sensing capacity of $M_{i}$ exactly at the position $p_{i}$ of UAV $A_{i}$. That is, we have
  (3) $$D_{i}(q_{i},q_{i})=M_{i}>D_{i}(q,q_{i}),\;\forall q\neq q_{i}$$

**Figure 1:**
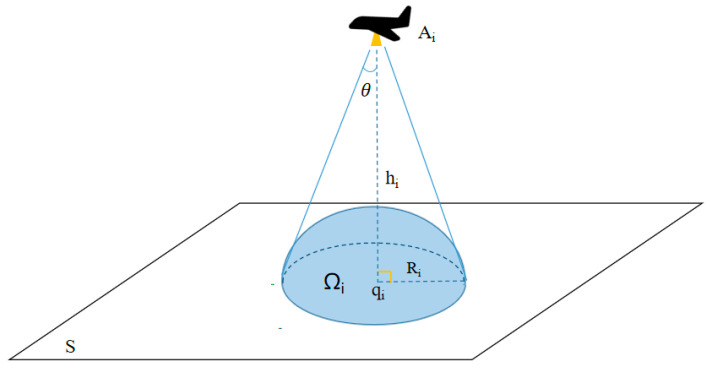
Diagram of the sensing region.

Hence, the divergence angle of the detector is θ, the detection height is *h_i_*, then, the sensing radius is *R_i_*, as shown in Figure 1.

(5) Mathematically, such a sensor function is a second-order polynomial function of $d=|q_{i}-q|$. Hence, the perceptual ability at the different position *q* of the UAV can be given by
  (4) $$G_{i}(d)=\left\{\begin{array}{cc} \frac{M_{i}}{R_{i}^{4}}{\left(d^{2}-R_{i}^{2}\right)}^{2} & ,d\leq R_{i}^{}\\ 0 & ,d>R_{i}^{} \end{array}\right.$$

The information accumulation of a point in the fire field obtained by a UAV using the sensor increases with time, that is, the coverage value of a point in the area increases with time. The coverage level of any point $q\in \Omega_{i}$ from the initial time t=0 to *t* is defined as follows:

(5) $$\gamma_{i}(q,t)=\int_{0}^{t}G_{i}(|q_{i}-q|)d\tau$$

Let k⊆I be the subset of UAVs that covered *q* and the effective coverage by a subset of UAVs $A_{k}=\left\{A_{i}|i\in k\subseteq I\right\}$ in surveying q is then given by [26,27]

(6) $$\gamma_{k}(q,t)=\int_{0}^{t}\sum_{i\in k}G_{i}(|q_{i}(\tau )-q{|}^{2})d\tau$$

It can be obviously seen that $\gamma_{k}(q,t)$ is a non-decreasing function of time. That is,

(7) $$\frac{\partial }{\partial t}\gamma_{k}(q,t)=\sum_{i\in k}G_{i}(|q_{i}(\tau )-q|)\geq 0$$

## 3. Adaptive Control Law

In the process of solving the second problem, the traditional artificial potential field method was abandoned. Because the acceleration of UAVs is limited, they need time and space to brake and avoid collisions. Therefore, the alignment interaction range was determined based on the expected optimal relation between the velocity difference and distance. Consequently, the movement state of each UAV itself can be adjusted in real time to effectively avoid collisions according to the relative distance and relative speed of the surrounding objects.

The rationale of adaptive control law is to use the flocking aggregation morphology control algorithm to enable the UAV group to construct a local network. The perception range is expanded to avoid collisions through the information interaction between adjacent UAVs. In addition, the gradient descent method is used to optimize the navigation path in real time to achieve the autonomous coverage and deployment of the UAV group to the fire field.

When an agent moves in the fire field, it will be affected by the driving force from the unknown area, the repulsive force from the obstacles or other adjacent agents, and the viscous force to reduce the difference in the velocity vectors of nearby UAVs or obstacles. So, the optimal velocity of an agent is the sum of the vectors of the optimum coverage velocity, repulsive velocity, and viscous velocity. The specific design process and implementation steps are as follows:

### 3.1. Optimum Coverage Velocity

The rationale of the optimum coverage velocity is to make each UAV move toward the direction of a low coverage level by establishing the potential energy function regarding the error between the expected coverage level and the actual coverage level, so that UAVs can autonomously and intelligently complete the coverage deployment task of mobile base stations.

Let $C_{0}$ be the desired attained effective coverage at all points in the S. The goal is to attain a network coverage of $\gamma_{k}(q,t)=C_{0}$ for all points q∈S at some time *t*. Consider the following error function:

(8) $$J(t)=\int_{\Omega }h(C_{0}-\gamma_{I}(q,t))dq$$

where h(x) is a penalty function, which is strictly convex in the interval (0,*C*_0_], twice differentiable, and positive definite, that is, $h(x)>0,h^{\prime }(x)>0$, $h^{\prime \prime }(x)>0,\forall x\in \left(0,C_{0}\right]$. And that satisfies $h(x)=h^{\prime }(x)=h^{\prime \prime }(x)=0$ for all x≤0. When J(t)=0, it means that the coverage task in the sensing area Ω is completed.

To guarantee the coverage of the entire domain *S* with an effective coverage of $C_{0}$ under appropriate assumptions, the UAV has to move toward the area where $\gamma_{I}(q,t)<C_{0}$. In order to improve the coverage efficiency, the area coverage problem can be described as the optimization problem of seeking the minimum J(t) in Formula (9) [28,29]:

(9) $$\mathrm{Minimize}\;J(t)=\int_{S}h(C_{0}-\gamma_{I}(q,t))dq,\;q_{i}\in S$$

The gradient descent method can be used to solve (9) to obtain the optimal coverage speed of the UAV. The accumulated error will generate an attractive “force” on a UAV. Once $\gamma_{I}(q,t)=C_{0}$ at a point, the error at this point is zero no matter how much additional time UAVs spend detecting that point, that is, the excessive coverage has no effect on the motion. Therefore, the optimal coverage speed of the UAV can be designed as follows:

(10) $$\begin{array}{ll} {\vec{v}}_{i1}^{}(t) & =-k_{c}\cdot \frac{dJ(t)}{dt}\\ & =-k_{c}\cdot \int_{S}h^{\prime }(C_{0}-\gamma_{I}(q,t))\sum_{i\in I}G(|q_{i}-q|)dq \end{array}$$

where $k_{c}>0$ is the fixed coverage velocity adjustment gain.

Since the optimal coverage speed of the UAV is based on local information, when the points in the perception area of the UAV are effectively covered, the UAV will tend to be stationary. If the whole area has not been covered effectively at this point, a traction speed is required for the UAV to avoid becoming stuck in a local minimum.

Firstly, set P(t) represents a set of points that are not effectively covered in the area S at time *t*. That is

(11) $$P(t)=\{q\in S:0<\gamma_{I}(q,t)<C_{0}\}$$

For UAV $A_{i}$, let $P_{i}(t)$ be the set of points with the shortest distance from $q_{i}(t)$ in set P(t), that is

(12) $$P_{i}(t)=\{q^{*}\in P(t):q^{*}=argmin_{q\in P(t)}|q_{i}-q|\}$$

A rule can be set to choose a unique point from the set to calculate the speed of the UAV at the next moment. The traction speed ${\vec{v}}_{s}$ of the UAV can be designed according to the artificial potential field method [30] as follows:

(13) $${\vec{v}}_{s}^{}=-k_{s}\cdot ({\vec{q}}_{i}-{\vec{q}}_{m})$$

where $k_{s}>0$ is the traction velocity adjustment gain, ${\vec{q}}_{i}$ is the position vector of the UAV projected on the OXY plane, and ${\vec{q}}_{m}$ is the position vector of point $q_{m}$ on the OXY plane.

In summary, the expected optimal coverage velocity of each UAV is:

(14) $${\vec{v}}_{i}^{c}(t)=\left\{\begin{array}{cc} \begin{array}{l} -k_{c}\cdot \frac{dJ(t)}{dt}\\ -k_{s}\cdot ({\vec{q}}_{i}-{\vec{q}}_{m}) \end{array} & \begin{array}{l} ,\;J(t)\neq 0\\ ,\;J(t)=C_{0}\neq 0 \end{array} \end{array}\right.$$

### 3.2. Repulsion

A linear velocity term was chosen for the local repulsion. Let $r_{0}^{}$ be the maximum interaction range under which the UAV starts to repulse other UAVs and obstacles:

(15) $${\vec{v}}_{ij}^{r}=\left\{\begin{array}{l} p_{0}^{}\cdot (r_{0}^{}-r_{ij})\cdot \frac{{\vec{r}}_{i}-{\vec{r}}_{j}}{r_{ij}}\;\begin{array}{cc} , & r_{ij}<r_{0}^{} \end{array}\\ 0\;,\;\mathrm{otherwise} \end{array}\right.$$

In the equation above, $p_{0}^{}$ is the linear gain of the pairwise repulsion and $r_{ij}=|{\vec{r}}_{i}-{\vec{r}}_{j}|$ is the distance between UAV i and obstacle *j*. The total repulsion term calculated for UAV i with respect to the other UAVs or obstacles is

(16) $${\vec{v}}_{i}^{r}=\sum_{j\neq i}{\vec{v}}_{ij}^{r}$$

where j is iterated for all other UAVs and obstacles.

### 3.3. Viscous Velocity

The pairwise velocity alignment can be obtained with a velocity term that depends on the difference in the velocity vectors of nearby UAVs or obstacles. Previous works based on the artificial potential field method adjusted the velocity of the UAV attenuating asymptotically to zero at the expected position according to the distance relative to the obstacle at a different time [31]. These models work fine when UAVs move within a lower velocity regime. However, the UAVs have to fly through the narrow corridor at a relatively high velocity in order to reduce the rescue time for people in distress at the high-rise fire scene. In this case, the previous method caused the obvious self-excited oscillations of the UAV in the process of the repulsion. Therefore, the viscous velocity appended to the control law was useful to solve this problem. It not only synchronizes motion to achieve the collective behavior of UAVs, but it also has to serve as a damping medium, reducing the self-excited oscillations emerging due to the relatively high velocity of the UAV during the repulsion.

To fulfill the practical deployment requirements of mobile base stations in the high-rise fire field, we have chosen an ideal braking function in the space that is denoted by D(.):

(17) $$D(r,a,p)=\left\{\begin{array}{l} 0\;\;\mathrm{if}\;r\leq 0\\ rp\;\;\mathrm{if}\;0\;<\;rp\;<\;a\;/\;p\\ \sqrt{2ar-a^{2}/p^{2}}\;\mathrm{otherwise} \end{array}\right.$$

where r is the distance between a UAV and an expected stopping point, a is the preferred acceleration, and p is a linear gain also determining the crossover point between the two phases of deceleration.

The rationale behind our viscous velocity term is to prohibit two UAVs having a larger velocity difference at a given distance than what is allowed by this ideal braking function and to serve as a buffer medium to eliminate the self-excited oscillations of the UAV:

(18) $$v_{ij}^{f\max}=\max(v^{f},D(r_{ij}-r_{0}^{f},a^{f},p^{f})),$$

(19) $$\begin{array}{l} {\vec{v}}_{ij}^{f}=\left\{\begin{array}{l} F_{s}(v_{ij}^{}-v_{ij}^{f\max})\cdot \frac{{\vec{v}}_{i}-{\vec{v}}_{j}}{v_{ij}^{}}\;\mathrm{if}\;v_{ij}^{}>v_{ij}^{f\max}\\ 0\;\;\mathrm{otherwise} \end{array}\right. \end{array}$$

In the equations above, $F_{s}^{}$ is a linear coefficient of the velocity alignment error reduction, $v_{}^{f}$ is a velocity slack to allow for a certain amount of velocity difference independently of the inter-UAV distance, $r_{0}^{f}$ is the distance of the stopping point for UAV i relative to and in front of UAV j, $p_{}^{f}$ and $a_{}^{f}$ are the linear gain and the acceleration parameters of the pairwise alignment, and $v_{ij}=|{\vec{v}}_{i}-{\vec{v}}_{j}|$ is the amplitude of the velocity difference between UAVs i and j. The total viscous velocity term calculated for UAV i with respect to the other UAVs—similarly to the repulsion term—is

(20) $${\vec{v}}_{i}^{f}=\sum_{j\neq i}{\vec{v}}_{ij}^{f}$$

where j is iterated for all other UAVs.

## 4. Simulation Results

In this section, we provide the simulation results for the fully connected network case. Assume that S is a square region with the side length of l=700 dm. Let the original position of each UAV be distributed randomly in the zone so that x∈[0,150] and y∈[550,700]. Let the original velocity of each UAV be $|{\vec{v}}_{0}^{}|=10\;\mathrm{dm}/s$ and set the direction of the original velocity be set randomly. Set the original relay distance $d_{r}=50\;\mathrm{dm}$. Let the effective coverage $C_{0}=40$ and the number of UAVs N=78. Obstacles are marked with the black color. The UAV is marked with the blue color and the base station is marked with the red color. The red line between two base stations means the distance between them $d_{b}\leq d_{r}$.

**Figure 2:**
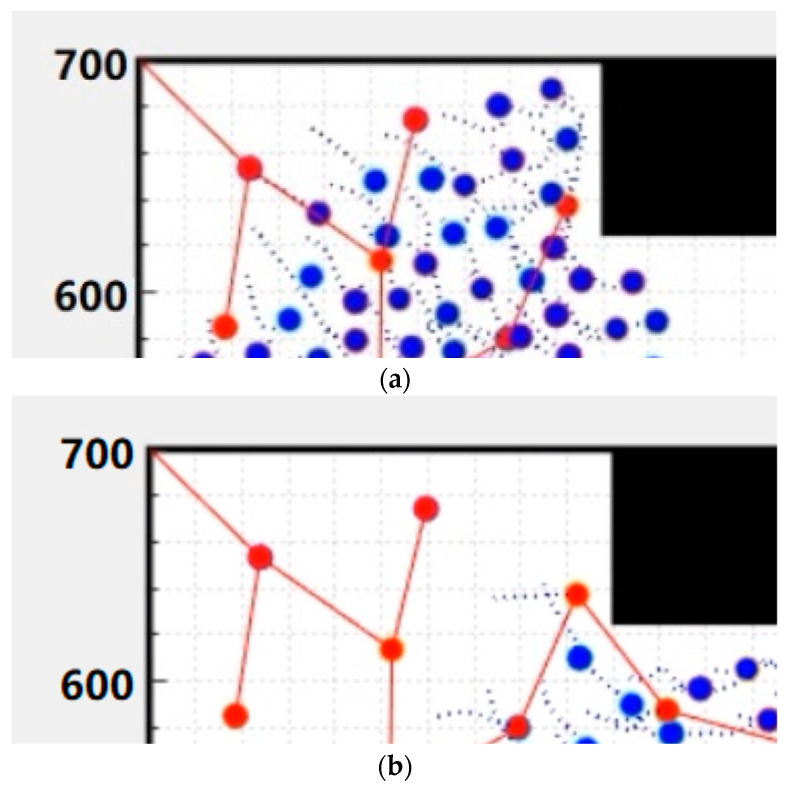

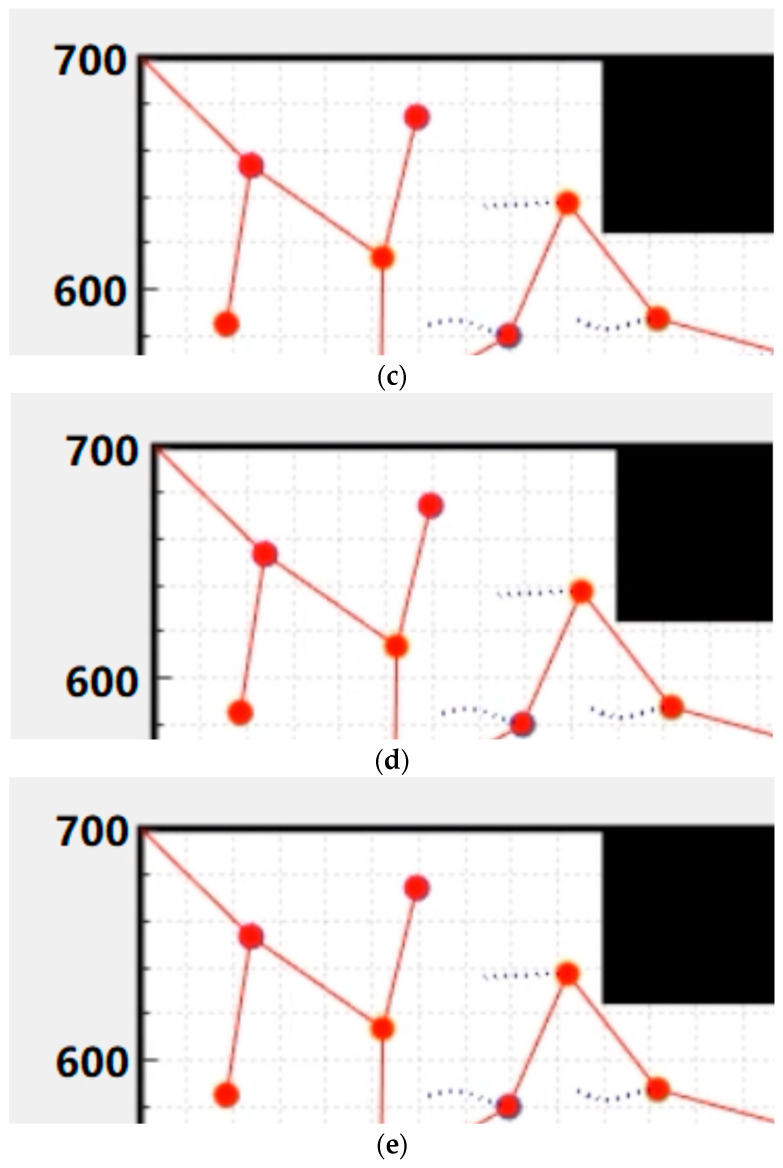
Coverage deployment of mobile base stations at different times: (**a**) coverage deployment at the time of 10 s; (**b**) coverage deployment at the time of 20 s; (**c**) coverage deployment at the time of 40 s; (**d**) coverage deployment at the time of 50 s; (**e**) coverage deployment at the time of 60 s.

Due to the fire field being an unknown complex environment, there is no way to plan the path for each UAV before the beginning of the mission. The only way is to depend on their cooperation. It can be seen from the trajectory of UAVs at different times in Figure 2 that each UAV can keep a safe distance from obstacles and other UAVs. There is no one UAV which collides with the obstacles and beyond the boundaries. It can be seen from Figure 2e that the base stations were deployed evenly in the unknown mission area with obstacles and can cover the whole mission area. There is no obstacle in the middle of the line between every two adjacent base stations so that the non-line-of-sight problem in the unknown complex environment can be turned into the line-of-sight problem. Therefore, the efficiency and accuracy of the target positioning will be greatly enhanced.

**Figure 3:**
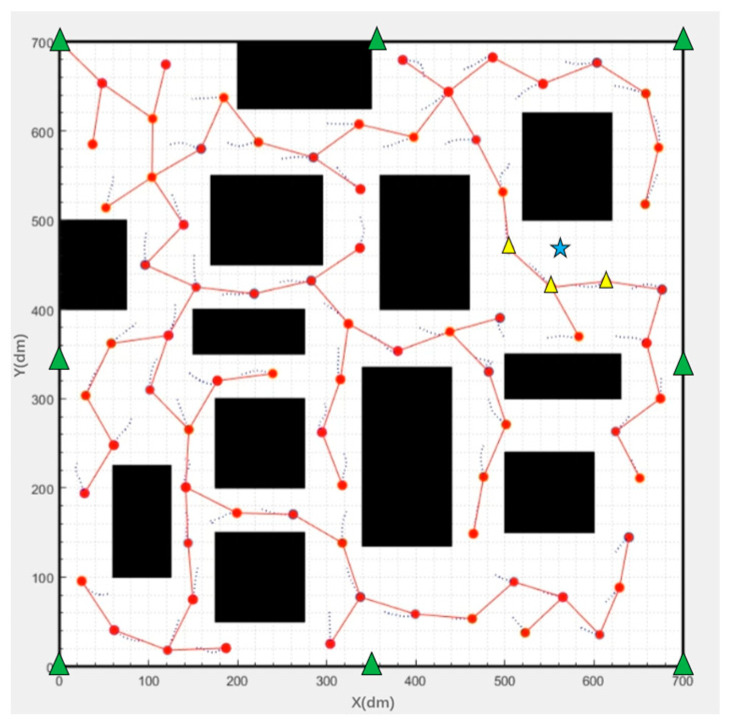
Diagram of base stations’ deployment of two algorithms.

**Figure 4:**
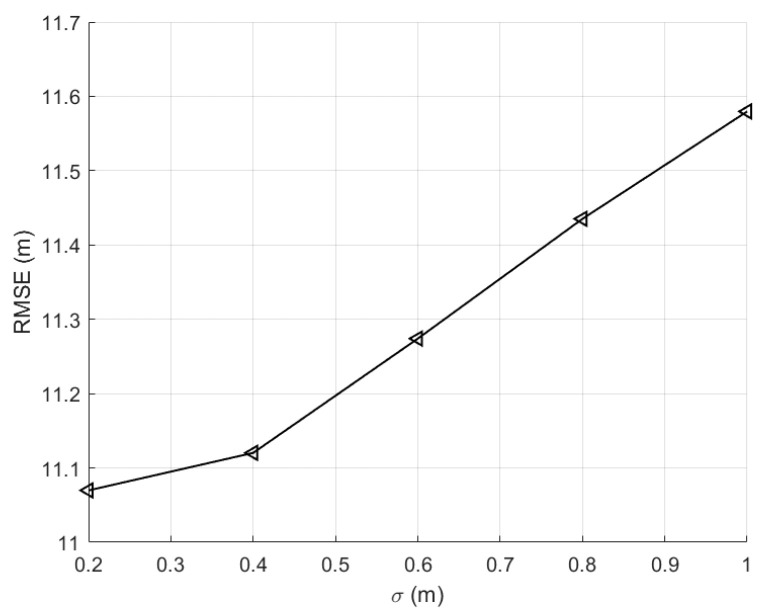
NLOS positioning error simulation results with eight base stations.

At present, the commonly used positioning method is to set a UWB positioning base station at the periphery of the fire field. We assume that eight UWB base stations were deployed evenly on the boundary of the fire field as shown by the green triangle mark in Figure 3 and the inter distance of adjacent UWB base stations is 35 m. There are more than three LOS positioning base stations nearby at any point in the fire field after the deployment of mobile base stations in the fire field is finished. The position of the firefighter is (550, 480) shown as the blue star mark in Figure 3 The distance between the firefighter and UWB base station can be obtained due to the UWB label on the firefighter. The closest three mobile base stations to the firefighter are marked by the yellow triangle in Figure 3 and were used to obtain the precise position information of the firefighter. It can be easily seen from Figure 4 that the problem for the common method is to solve the NLOS positioning, but the problem for the method in this paper is to solve the LOS positioning.

According to our experience, we assume that the system error of the UWB is 0.2 m, 0.4 m, 0.6 m, 0.8 m, and 1 m, respectively. The position of the firefighter was measured many times with the same UWB unit. The optimal position of the firefighter was estimated with the least square method [32,33]. The simulation results of the above two methods are shown in the following figures.

## 5. Discussion

The most important problem in the deployment of mobile base stations for coverage with connectivity constraints is how to control their movement and behavior to achieve a desired configuration and how to establish decentralized coordination among the multiple UAVs team. This becomes more difficult for the navigation and positions of UAVs in the absence of GPS. In the absence of GPS, robots can only perform local localization by estimating their adjacent UAV’s relative locations and sending limited messages out with Zigbee. Especially in the fire field of a high-rise building, this mechanism is more significant to realize the fast deployment of UWB base stations with a UAV group.

**Figure 5:**
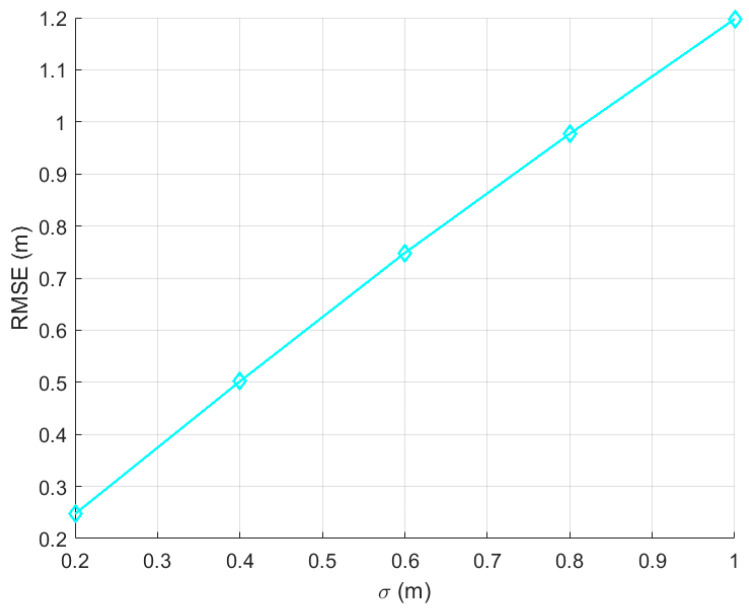
LOS positioning error simulation results with three base stations.

In the simulation experiment, the system error of the UWB is set as 0.2 m, 0.4 m, 0.6 m, 0.8 m, and 1 m, respectively. It can be seen from Figure 5 that the positioning error of the algorithm presented in this paper is less than 1.2 m. It is much less than the positioning error of the traditional algorithm that is more than 10 m, as shown in Figure 4.

On the other hand, the large scale of a multi-agent system can effectively reduce the moving distance of each agent. The less moving distance not only reduces the accumulative positioning error but also means low energy a large scale of smaller and cheaper UAVs can be used to satisfy the requirement for the deployment of mobile UWB base stations in the fire field.

The real-world implementation of such a system could face potential challenges. The most important one is that the size, shape, and position of the obstacle would change randomly with the development of the fire. Therefore, the self-adaptive control law of the multi-agent system is a very challenging issue that is worth further research.

## 6. Conclusions

In this paper, we formulated a control problem that addresses the autonomous deployment of a massive multi-agent system in a complex unknown high-rise fire environment. The goal is to achieve the coverage deployment of mobile base stations to make sure that there are more than three base stations nearby to any one point in the fire field so that the NLOS positioning problem of the common positioning method can be transformed into the LOS positioning problem. The viscous velocity was appended to the control law. The simulation results show that the coverage deployment of the mobile base stations can be realized safely in the complex environment with unknown obstacles. Finally, we compared the simulation results of the common method and the method illustrated in this paper. The result of the comparison shows that this method can greatly improve the positioning accuracy and meet the practical positioning requirements of a high-rise fire rescue.

## Data Availability

Where no new data were created.
